# Supplementary material for: Characterization of Food Packaging Films with Blackcurrant Fruit Waste as a Source of Antioxidant and Color Sensing Intelligent Material
Source: Molecules. 2021 Apr 28;26(9):2569. doi: 10.3390/molecules26092569 (PMC8124232; doi:10.3390/molecules26092569)
Supplement: Supplementary file 1 [file molecules-26-02569-s001.zip › molecules-1194733-supplementary.pdf]

molecules

MDPI

Supplementary Material

Characterization of Food Packaging Films with Blackcurrant Fruit Waste as a Source of Antioxidant and Color Sensing Intelligent Material

Mia Kurek [1,*], Nasreddine Benbettaieb [2,3], Mario Ščetar [1], Eliot Chaudy [2], Maja Repajić [1], Damir Klepac [4,5], Srećko Valić [4,5], Frédéric Debeaufort [2,3] and Kata Galić [1]

[1] Faculty of Food Technology and Biotechnology, Pierottijeva 6, 10000 Zagreb, Croatia; mscetar@pbf.hr (M.Š.); maja.repajic@pbf.unizg.hr (M.R.); kgalic@pbf.hr (K.G.)
[2] Department of BioEngineering, Institute of Technology, University of Burgundy, 7 Blvd Docteur Petitjean, BP 17867, F-21078 Dijon, CEDEX, France; nasreddine.benbettaieb@u-bourgogne.fr (N.B.); chaudy.eliot1@gmail.com (E.C.); frederic.debeaufort@u-bourgogne.fr (F.D.)
[3] UMR PAM A 02.102, Agrosup Dijon, Univ. Bourgogne Franche-Comté, F-21000 Dijon, France
[4] Faculty of Medicine, University of Rijeka, Braće Branchetta 20, 51000 Rijeka, Croatia; damir.klepac@medri.uniri.hr (D.K.); svalic@medri.uniri.hr (S.V.)
[5] Centre for Micro- and Nanosciences and Technologies, University of Rijeka, Radmile Matejčić 2, HR-51000 Rijeka, Croatia
* Correspondence: mkurek@pbf.hr; Tel.: +385-1460-5003

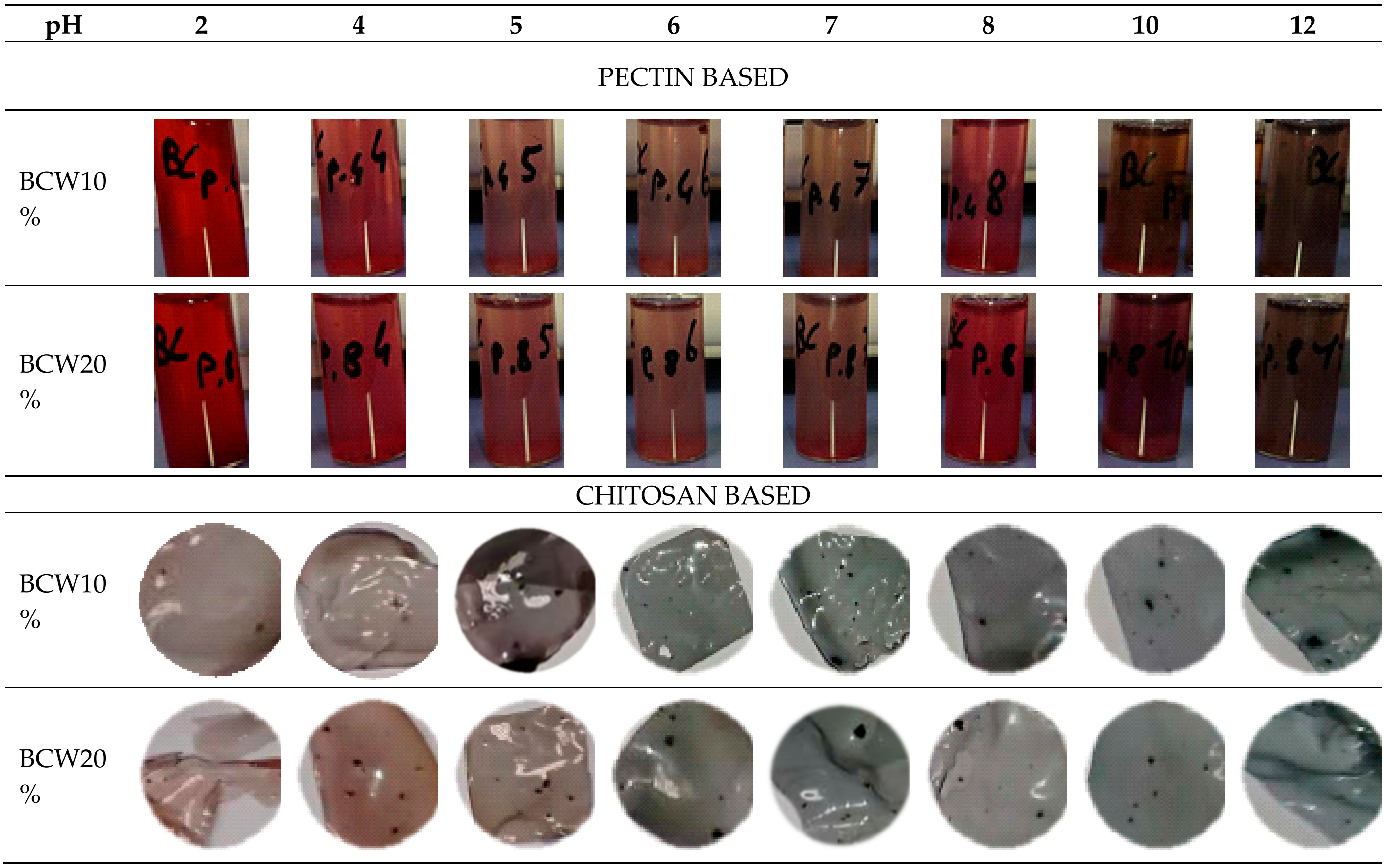

Figure S1. Photos of chitosan and pectin films with blackcurrant powder (BCW) (10 and 20%).

Table S1. FTIR spectral data (ν –stretching: as-asymmetrical, s-symmetrical, δ -deformation) of chitosan (CS) and pectin (PEC) without or with blackcurrant powder (BCW).

**Film**	**ν O-H, ν NH_2**	**ν_{as} C-H**	**ν_{s} C-H**	**ν C=O (amide I)**	**δ NH (amide II)**	**δ C-H**	**ν_{s} C-O-C**	**ν C-O**	**ν C-O**
CHITOSAN									
CS	3647-2968	2929	2879	1651	1546	1406	1151	1063	1019
CS10BCW	3688-2981	2929	2881	1650	1543	1406	1151	1064	1019
CS20BCW	3676-2975	2930	2881	1650	1543	1406	1151	1064	1019
PECTIN	**ν O-H**	**ν C-H**	**ν COOH**		**ν C=C**	**ν C-O**			
PEC	3689-2993	2934	1744		1638	1014			
PEC10BCW	3674-2979	2937	1740		1638	1015			
PEC20BCW	3689-3000	2937	1735		1638	1014			

CS-chitosan, CS10BCW and CS20BCW-chitosan with 10 and 20% (*w*/*w*) blackcurrant powder, PEC-pectin. PEC10BCW and PEC20BCW-pectin with 10 and 20% (*w*/*w*) blackcurrant powder.
